# Evaluation of preoperative ultrasonographic and biochemical features of patients with aggressive parathyroid disease: is there a reliable predictive marker?

**DOI:** 10.1590/2359-3997000000224

**Published:** 2016-11-07

**Authors:** Bekir Cakir, Sefika Burcak Polat, Mehmet Kilic, Didem Ozdemir, Cevdet Aydin, Nuran Süngü, Reyhan Ersoy

**Affiliations:** 1 Yildirim Beyazit University Ataturk Education and Research Hospital Ankara Turkey Yildirim Beyazit University, Ataturk Education and Research Hospital, Endocrinology and Metabolism Department, Ankara, Turkey; 2 Yildirim Beyazit University Ataturk Education and Research Hospital Ankara Turkey Yildirim Beyazit University, Ataturk Education and Research Hospital, General Surgery Department, Ankara, Turkey; 3 Yildirim Beyazit University Ataturk Education and Research Hospital Ankara Turkey Yildirim Beyazit University, Ataturk Education and Research Hospital, Pathology Department, Ankara, Turkey

**Keywords:** Atypical parathyroid adenoma, parathyroid carcinoma, biochemical markers, ultrasound

## Abstract

**Objective:**

Parathyroid cancer (PC) represents < 1% of cases of PHPT. Tumors demonstrating atypical histopathologic features and don’t fulfill criteria for carcinoma are classified as atypical adenomas (APA). The purpose of this study was to determine a biochemical or ultrasonographic feature that can predict aggressive disease requiring more extensive surgery and closer follow-up.

**Subjects and methods:**

Twenty eight patients operated for PHPT and diagnosed with atypical adenoma (23 patients) or carcinoma (5 patients) were enrolled in this study. The control group consisted of 102 patients operated between the same dates and diagnosed with classical PA. Classical adenomas, atypical adenomas, and carcinomas were compared according to their biochemical and ultrasonographic parameters.

**Results:**

Serum Ca levels were significantly higher in the PC group compared with the APA and classical PA groups. Serum median PTH, Serum ALP and UCa was significantly higher in the APA and carcinoma groups compared to the classical PA group. ROC analysis was made to determine the best cut off values for predicting aggressive disease were 12.45 mg/dL, 265.05 pg/mL, 154.5 IU/l, 348.5 mg/day and 21.5 mm for Ca, PTH, ALP, UCa and the adenoma diameter, respectively. Multivariate analysis showed that serum Ca, ALP and isoechoic/cystic appearance were independent predictors for aggressive disease.

**Conclusion:**

Preoperatively high PTH, ALP, and UCa levels and large lesions with isoechoic or cystic appearances may be predictive of atypical adenoma or carcinoma in patients being evaluated for PHPT. In such cases, surgeons may prefer en bloc parathyroidectomy to minimally invasive surgery.

## INTRODUCTION

Primary hyperparathyroidism (PHPT) is one of the most common endocrine diseases. It is usually caused by a single lesion; however, occasionally, there may be multiple tumors in the parathyroid glands. Cancer is rarely seen and represents 0.005% of all cancers and < 1% of cases of PHPT in the United States. It affects men and women equally, and its management and outcome are variable (1,2).

Although parathyroid carcinoma (PC) has a characteristic intraoperative gross appearance, including invasion to neighboring anatomical structures and being densely firm, the final diagnosis is made upon histopathological examination, in which an invasive growth pattern or metastasis must be demonstrated. The World Health Organization (WHO) morphological criteria are currently used for diagnosis. Extensive invasion of adjacent tissues and metastatic spread represent the two absolute diagnostic criteria (3). The other criteria include focal coagulation necrosis, irregular dense fibrosis, and capsular, vascular, or neural invasion. The presence of certain cytological and architectural features such as adherence to adjacent organs, a solid growth pattern, broad bands of fibrosis, cytological atypia, and an irregular growth contour do not indicate malignancy but are recognized as atypical features encountered more commonly in malignant than benign tumors. Tumors that demonstrate these atypical features and do not fulfill criteria for carcinoma can be classified as atypical parathyroid adenomas (APA) (4,5). It is postulated that atypical adenomas may be a preceding stage of carcinoma development, and this entity is expected to include tumors previously referred to as equivocal carcinomas (6). The clinical importance and long-term outcomes as well as appropriate operative management and surveillance are not well defined for APA probably due to the overall low prevalence as well as the lack of a standard definition of APA. For aggressive parathyroid tumors, early diagnosis and a radical therapy approach are paramount if clinical suspicion is made prior to surgery.

There is no single biochemical parameter or imaging property that can distinguish aggressive parathyroid disease (APA and carcinoma) from classic adenomas, and scarce data exist in the literature concerning any suspicious ultrasonographic features that can predict it (7).

Herein, we aimed to evaluate the preoperative imaging and biochemical features of patients histopathologically diagnosed with APA or carcinoma. The purpose of this study was to determine a biochemical or ultrasonographic feature that can predict aggressive disease requiring more extensive surgery and closer follow-up to prevent any lifelong complications.

## SUBJECTS AND METHODS

A hundred and sixty two patients were operated for PHPT from January 2011 to September 2015 in our center. Thirty two patients were excluded from the study. Exclusion criteria were; having chronic renal failure (4 patients), secondary or tertiary hyperparathyroidism (2 patients), familial syndromes (1 with MEN1 and 2 with MEN2A) and incomplete patient records (23 patients). Finally twenty eight patients diagnosed with atypical adenoma or carcinoma (23 patients with APA and 5 with PC) and a hundred and two patients operated between the same dates and diagnosed with classical PA with complete histopathology and preoperative patient records were enrolled in this study.

The study was approved by the local ethics committee of the University Hospital. Tumors were classified as either classical parathyroid adenoma (PA) or aggressive parathyroid tumors (APA or PC). APA was defined by the presence of two of the following characteristics: clinical/intraoperative adherence, bands of fibrosis, pronounced trabecular growth, and mitotic rates of > 1/10 high-power fields (hpfs) (4,5). APA also lacked any of the indisputable criteria for malignancy, including invasion of vascular or perineural soft tissue, or surrounding structures (including the thyroid, recurrent laryngeal nerve, trachea, and esophagus) or documented metastatic disease. Insufficient clinical data or missing histopathological reports were defined as exclusion criteria. All patients’ demographic, clinical, and biochemical characteristics were reviewed, with an emphasis on collection of parathyroid disease-specific characteristics. Biochemical data included preoperative serum calcium (Ca, mg/dL), phosphorus (P, mg/dL), parathyroid hormone (PTH, pg/mL), and alkaline phosphatase (ALP, IU/L) levels, and 24 h urinary calcium excretion (UCa, mg/day) and they were collected from the records immediately prior to surgery in all patients. Reference ranges for Ca, P, PTH, ALP, and 24 h UCa were 8.5–10.5 mg/dL, 2.5–4.5 mg/dL, 15-60 pg/mL, 36–113 IU/L, and 25–300 mg/day, respectively.

Bone mineral density (BMD) was measured by DXA (QDR-4500, Hologic Inc, Waltham, MA) at the lumbar spine in posterior-anterior projection (L1-L4) (LS BMD) and femoral sites [femoral neck (FN-BMD)]. One-third distal non dominant forearm measurement was not available in most of the patients. BMD was expressed as T-score or Z-score. T-scores was taken into consideration in postmenopausal women and males older than 50 years of age while Z scores were evaluated in premenopausal women and young males.

**Figure 1:**
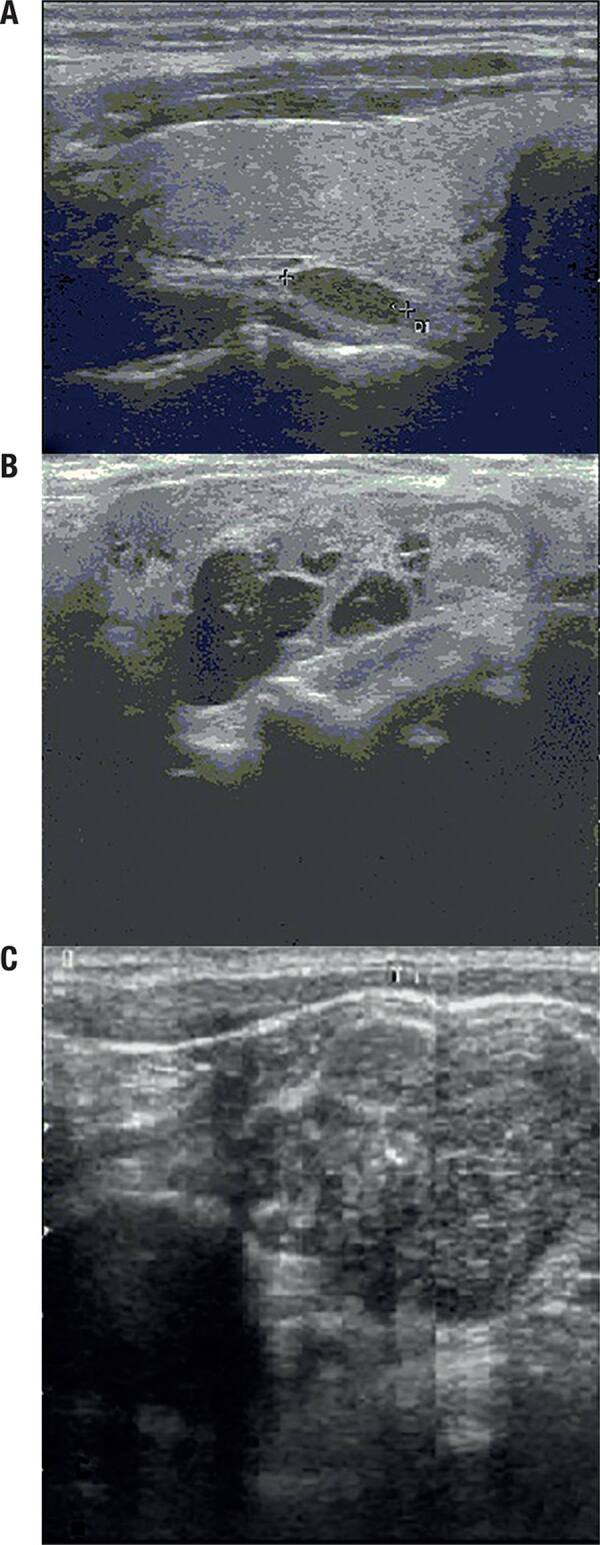
Hypoechoic, cystic and isoechoic appearances of the lesions belonging to a classical adenoma, carcinoma and atypical adenoma, respectively.

Imaging data included reports of preoperative ultrasound (US) and sestamibi scans. US was performed by experienced endocrine specialists. All lesions were localized by US in the APA and PC groups whereas all except 4 lesions were localized in the classical PA group. Lesions were recorded as isoechoic, hypoechoic, or cystic according to their ultrasonographic appearance (Figure 1). Sizes and the localization of the lesions were recorded. Classical adenomas, atypical adenomas, and carcinomas were compared according to their biochemical and ultrasonographic parameters.

### Statistical analysis

Data analysis was performed using the SPSS version 17.0 software (IBM Corporation, Armonk, NY, USA). For all data, the assumption of normal distribution was verified using a Kolmogorov-Smirnov test. Levene’s test was used for the evaluation of homogeneity of variances. Continuous data are shown as the mean ± standard deviation (SD) or the median and interquartile range, and categorical variables are shown as the number of individuals and percentages.

The mean differences among groups were analyzed by one-way analysis of variance (ANOVA). The Kruskal Wallis test was used for comparisons of medians, and the Conover’s multiple comparison test was used for *post hoc* comparisons. Categorical data were evaluated using the chi-square test or Fisher’s exact test, where appropriate.

The area under the receiver operating characteristic (ROC) curve (AUC) and 95% confidence intervals (CI) for both laboratory and adenoma diameter measurements were calculated. When the AUC was statistically significant, the optimal cut off point of each predictor was determined as the value giving the maximum sum of sensitivity and specificity. The diagnostic performances of laboratory and adenoma diameter measurements were evaluated including sensitivity, specificity, positive predictive value (PPV) and negative predictive value (NPV).

Multiple binary logistic regression with the backward method was used to determine the best predictor(s) that affected diagnosis. Any variable whose univariate test had a p value < 0.05 was included in the multivariate model. Odds ratios, 95% CIs, and Wald statistics were also calculated for each variable.

All variables were considered significant at a value of p < 0.05. The Bonferroni correction was applied to multiple comparisons to control for type I errors.

## RESULTS

After exclusion of 32 patients (4 with CKD, 23 with incomplete patient records, 2 with tertiary hyperparathyroidism, 1 MEN1 with parathyroid hyperplasia and 2 MEN2A), data from 130 patients were analyzed. There were no significant differences between the groups with regard to age (p = 0.991). The sex distribution was significantly different between the classical PA and the APA groups; there were significantly more females in the classical PA group, whereas there were significantly more males in the APA group (p < 0.05). Serum Ca levels were significantly higher in the PC group compared with the APA and classical PA groups (p < 0.001 and p = 0.010, respectively). Serum P levels did not differ between the groups (p = 0.370) while PTH levels were significantly higher in the APA and PC groups compared with the classical PA group (p < 0.05). Serum ALP and 24 h UCa excretion were significantly higher in the APA and PC groups compared with the classical adenoma group (p < 0.001). 25-hydroxy vitamin D levels were not significantly different between the groups. Serum creatinine levels were not significantly different between the groups. FN-BMD expressed as T/Z scores were significantly lower in APA and PC groups compared with the classical PA group whereas LS-BMD scores did not differ significantly.

The largest adenoma diameter measured by preoperative US was higher in the APA and PC groups than in the classical adenoma group (p < 0.001), and there were also significant differences between the three groups with regard to the ultrasonographic appearance of the adenomas. Classical PAs had a mostly hypoechoic appearance whereas APA and carcinomas had a more isoechoic or cystic appearance. There were no differences between the groups with regard to positive involvement in nuclear imaging (p = 0.158). As expected, disease recurrence/persistence and need for an additional surgical intervention were more prevalent in the PC group compared with the other two groups (p = 0.011). Demographic, biochemical, and imaging data are shown in Table 1.

**Table 1:** Comparison of the demographic, biochemical and imaging data of the patients in three histological subgroups

	Classic (n = 102)	Atypical (n = 23)	Carcinoma (n = 5)	p-value
Mean age (years)	51.1 ± 14.0	51.3 ± 13.7	50.4 ± 13.7	0.991^†^
Male/Female	20/80 (20.0-80%)^a^	11/12 (47.8-52.2%)^a^	3/2 (60.0-40%)	**0.006** ^**‡**^
Median Ca (mg/dL)	11.3 (9.2-14.7)^b^	11.6 (10-16)^c^	16.0 (11.6-16.5)^b,c^	**0.005** ^**¶**^
Median P (mg/dL)	2.5 (1.1-4.5)	2.3 (2.5-2.9)	2.1 (1.4-3.5)	0.370^¶^
Median PTH (pg/mL)	167.5 (60-900)^a,b^	448.0 (139-735)^a^	520.0 (163-1077)^b^	**< 0.001** ^**¶**^
Median ALP (IU/L)	101.0 (27-244)^a,b^	190.0 (66-718)^a^	589.0 (119-1880)^b^	**< 0.001** ^**¶**^
Median urine Ca (mg/day)	370.5 (68-948)^a,b^	500.0 (362.0)^a^	500.0 (483-900)^b^	**0.002** ^**¶**^
Mean 25-OH-Vit D (ng/mL)	13.47 ± 0.39	12.84 ± 0.47	11.57 ± 0.34	0.072
Median serum creatinine mg/dL	0.6 (0.4-1.2)	0.8 (0.5-1.8)	1.0 (0.6-1.9)	0.087
Median femur neck T score*	-1.06 (-3. 9-1.1)^a,b^	-1.90 (-3.2- 0.2)^a^	-2.8 (-3.7-0.1)^b^	**0.006**
Median lumbar spine T score*	-1.63 (-5.3-1.1)	-2.38 (-5.5-0.3)	-2.45 (-5.8-0.8)	0.13
Median lesion diameter (mm)	14.5 (5-43)^a,b^	22.0 (5-58)^a^	32.0 (15-44^)b^	**< 0.001** ^**¶**^
USG				**< 0.001** ^**‡**^
Hypoechoic	95 (95.0%)^a,b^	15 (65.2%)^a^	1 (20.0%)^b^	
Isoechoic	3 (3.0%)^a,b^	5 (21.7%)^a^	3 (60.0%)^b^	
Cystic	2 (2.0%)^a^	3 (13.0%)^a^	1 (20.0%)	
Positive MIBI	63 (65.6%)	18 (78.3%)	5 (100.0%)	0.158^‡^
Persistent/Recurrent disease	3 (3.0%)^b^	1 (4.3%)^c^	3 (60.0%)^b,c^	**< 0,001** ^**‡**^

† One-Way ANOVA; ‡ Chi-square test; ¶ Kruskal Wallis test; a: Classic vs Atypical (p < 0.05); b: Classic vs carcinoma (p < 0.05); c: atypical vs carcinoma (p = 0.010). * Z score was used for premenouposal females and males younger than 50 years of age.

**Figure 2:**
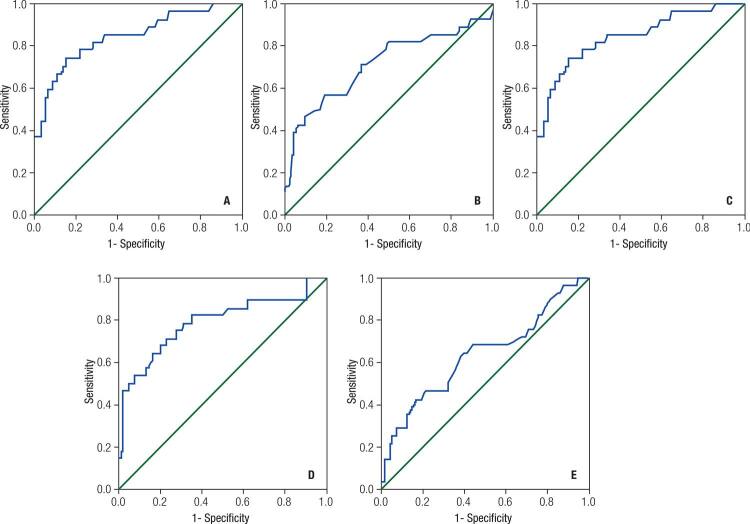
(A) ROC analysis for serum Ca (12.3 mg/dL cut-off had 42.9 sensitivity, 83% specificity, (B) ROC analysis for serum PTH (265.05 pg/mL cut-off had 71% sensitivity and 77% specificity), (C) ROC analysis for serum ALP (154.5 IU/L cut had 74.1% sensitivity and 84.6 specificity), (D) ROC analysis for Uca (348.5 mg/day cut off had 88.5% sensitivity and 47.8% specificity, (E) lesion size (21.5 mm had 57.1% sensitivity and 81.1% specificity).

Results of the ROC analysis investigating whether serum Ca, ALP, UCa, and PTH levels had statistically significant diagnostic value in differentiating classical adenomas from aggressive disease (APA or cancer) are shown in Table 2 and Figure 2. The AUC for serum Ca was statistically significant (AUC = 0.636, 95% CI: 0.509–0.762, p = 0.029) (Figure 2A). The best cut-off value for Ca for predicting aggressive disease was 12.45 mg/dL (sensitivity: 42.9%, specificity: 83.0%, PPV: 41.4%, NPV: 83.8%). The AUC for serum P was not statistically significant (AUC = 0.587, 95% CI: 0.450–0.724, p = 0.160), while that for PTH was significant (AUC = 0.787, 95% CI: 0.675–0.898, p < 0.001) and the best cut-off value for PTH was 265.05 pg/mL (sensitivity: 71.4%, specificity: 77%, PPV: 46.5%, NPV: 90.6%) (Table 2, Figure 2B). For serum ALP, the AUC was statistically significant (AUC = 0.843, 95% CI: 0.750–0.936, p < 0.001) and the best cut-off value was 154.5 IU/l for distinguishing aggressive disease from classical adenoma (sensitivity: 74.1%, specificity: 84.6%, PPV: 58.8%, NPV: 91.7%) (Table 2, Figure 2C). The AUC for 24 h UCa was statistically significant and the best cut-off value was 348.5 mg/day (sensitivity: 88.5%, specificity 47.8%, PPV: 32.4%, NPV: 93.6%) (Table 2, Figure 2D).

**Table 2:** Areas under curve for biochemical and ultrasonographic parameters for distinguishing classical adenomas from aggressive disease, best cut off points and diagnostic performances

	AUC	95 % CI	p-value	Cut-off	Sensitivity	Specificity	PPV	NPV
Ca	0.636	0.509-0.762	**0.029**	> 12.45	0.429	0.830	0.414	0.838
PTH	0.787	0.675-0.898	**< 0.001**	> 265.05	0.714	0.770	0.465	0.906
ALP	0.843	0.750-0.936	**< 0.001**	> 154.5	0.741	0.846	0.588	0.917
Urinary Ca	0.716	0.604-0.829	**< 0.001**	> 348.5	0.885	0.478	0.324	0.936
Lesion diameter	0.715	0.590-0.839	**< 0.001**	> 21.5	0.571	0.811	0.471	0.865

AUC: area under the curve; CI: confidence interval; PPV: positive predictive value, NPV: negative predictive value.

Among the ultrasonographic parameters, the AUC was statistically significant for the longest lesion diameter (AUC = 0.715, 95% CI: 0.590–0.839, p < 0.001) and the best cut-off value was 21.5 mm (sensitivity: 57.1%, specificity 81.1%, PPV: 47.1%, NPV: 86.5%) (Table 2, Figure 2E).

We performed a univariate analysis to predict risk factors for aggressive disease; variables with a p-value < 0.05 were subsequently included in a multiple logistic regression analysis. When the groups were controlled for all other possible risk factors, the most valuable parameters that predicted aggressive disease were serum Ca, ALP, and ultrasonographic appearance. The risk for aggressive disease increased 10.885-fold with values of ALP > 154.5 IU/L (95% CI: 3.116–37.810, p < 0.001) and 3.8-fold with values of Ca > 12.45 mg/dL (95% CI: 1.061–14.100, p = 0.040). Moreover, the risk for aggressive disease was 37.158-fold higher if the lesion had an isoechoic appearance rather than a hypoechoic appearance (95% CI: 3.027–456.126, p = 0.005). In addition, a cystic appearance also increased aggressive disease risk 11.644-fold compared with a classical hypoechoic appearance (95% CI: 1.023–132.504, p = 0.048). The results of the multivariate analysis are summarized in Table 3.

**Table 3:** Multivariate analysis of variables that can independently predict presence of aggressive disease

	Odds ratio	95% Confidence interval		Wald	p-value

		Lower	Upper		
Ca > 12.45 mg/dL	3.867	1.061	14.100	4.200	**0.040**
ALP > 154.5 IU/L	10.855	3.116	37.810	14.025	**< 0.001**
Hypoechoic	1.000	-	-	-	**-**
Isoechoic	37.158	3.027	456.126	7.984	**0.005**
Cystic	11.644	1.023	132.504	3.914	**0.048**

The mean follow-up period was 3.2 years and all PC patients had recurrence or metastasis during the follow-up. There was no recurrent disease in the APA or classical PA groups, although three patients in the APA group and one patient in the classical PA group had persistent disease (sustained high levels of serum Ca and PTH within six months of surgery) and required an additional surgery.

## DISCUSSION

In this report, we aimed to identify any preoperative biochemical and ultrasonographic variables that could differentiate classical adenomas from more aggressive parathyroid diseases, including APA and carcinomas.

The patient age did not differ significantly among patients with APA, classical PA, and PC in accordance to a previous report (8). However in many other series parathyroid carcinoma patients were younger than ones with benign adenomas (9). The sex distribution was different between the classical adenoma and APA groups, with a significant predominance of males in the APA group. The male-to-female ratio was also higher in the PC group, but was not significantly different from the classical adenoma group, which is most likely due to the low number of patients. Benign adenomas are most frequently seen in females. APA and carcinomas are similar in that the genetic bias that predisposes women to benign adenomas is not as dominant in APA and PC. This is supported by previously described sex-dependent patterns of chromosomal loss in men and women with PC (10).

PHPT is often associated with borderline or mild hypercalcemia (serum Ca < 11 mg/dL); values above 13 mg/dL are unusual in PHPT, although they do occur, and are more common in patients with parathyroid carcinoma-associated hypercalcemia (11). In our study, the median serum Ca level was significantly higher in the carcinoma group than in the APA and classical PA groups, similar to results of a previous report (12). All but one patient with PC had severe hypercalcemia, defined as serum Ca > 14 mg/dL. In a previous study with PC patients, serum Ca was identified as the most promising variable for the differentiation of patients with or without PC (13), whereas in another study, it was not determined to be an independent marker of carcinoma in a multivariate analysis (14). When we considered APA and PC patients together in the aggressive disease group, best cut-off value for Ca for predicting aggressive disease was 12.45 mg/dL. Thus, it was found to be an independent predictive marker for aggressive disease. To our knowledge, our study is the first to detect a cut-off value for distinguishing aggressive disease including APA and carcinomas.

PTH levels were significantly higher in the APA and PC groups compared to the classical PA group, but were not found to be an independent predictor for aggressive disease in the multivariate analysis, supporting the results of a previous report (13). The median PTH levels in the APA and carcinoma groups were similar, which supports a study by Fernandez-Ranvier and cols., which demonstrated no significant differences between the two groups with regard to PTH, although levels were slightly higher in the carcinoma group (15). However, McCoy and cols. demonstrated that PTH levels were significantly lower in patients with benign adenoma compared with PC, whereas they were similar between patients with benign adenoma and APA (16).

Serum ALP was significantly higher in the APA and PC groups than in the classical adenoma group; levels were not significantly different between the APA and PC groups. ALP was an independent predictive marker for aggressive disease, similar to a previous study in which serum ALP was significantly higher in carcinoma patients than in those with benign disease after all variables were adjusted (14). In our study, the best cut off value for ALP for distinguishing aggressive disease from classical adenoma was 154.5 IU/L (sensitivity: 74.1%, specificity: 84.6%). In the aforementioned study, the cut-off value of serum ALP was 285 IU/L and the sensitivity and specificity were 83.3% and 97.0%, respectively (14). Our cut-off value was lower, which was likely to be due to the inclusion of patients with atypical adenomas in the aggressive disease group, who had lower levels of ALP compared to carcinoma patients. We also detected that 24 h UCa excretion was significantly higher in the APA and PC groups than in the classical adenoma group, but it was not an independent predictor for aggressive disease. While interpreting that result it should be kept in mind that UCa is susceptible to the clinical condition and vitamin D status of the patient (17).

In our study most of the patients were vitamin D deficient. Epidemiological studies suggests that the prevalence of hypovitaminosis D (including deficiency and insufficiency) is more prevalent in patients with PHPT than in the general population regardless of sex, age and the season (18). The reason for that co-existence is not fully understood but there are several explanations in the literature such as increased conversion of 25 OH D to 1, 25 OHD by increased PTH (19). It is well established that hypovitaminosis D might result in increased PTH values and may mask a more significant evaluations in serum Ca, because of reduced Ca absorbtion. Increased PTH levels indeed aggravate bone catabolism, turnover and bone loss (20). In addition to that there is an increased risk of osteoporotic fractures in patients with PHPT in case of low vitamin D levels (21). There are controversies about the effect of vitamin D repletion on BMD at lumbar spine and femur neck. Some authors suggest that BMD is increased with repletion in both sites whereas others claim that 25 OHD has no independent effect on BMD (22,23). Besides the negative effects on BMD, low vitamin D levels may contribute to profound and lengthened hypocalcaemia (hungry bone syndrome) after parathyroidectomy (20). Therefore it has generally been recommended to supplement vitamin D to normalize 25(OH) vitamin D levels, although there are so far no available data to support the premise that this would contribute to the prevention of hypocalcaemia. In our center, we correct vitamin D deficiency aiming at serum levels > 20 ng/mL unless the patient has severe hypercalcaemia in the light of the current studies. There is no consensus about the optimum replacement dose and interval in the literature. In our unit, vitamin D repletion is undertaken using cholecalciferol 50.000 IU/ week for 1 month and vitamin D is checked monthly in order to adjust the maintenance dosage (1500-2000 IU/day).

In our study, femur neck T scores were significantly lower in APA and PC group compared with the classical PA group where as vertebra T scores didn’t differ significantly between the groups. It is known that in PHPT patients may have decreased BMD, in particular at more cortical sites (forearm and hip) as compared with more trabecular sites and the degree of bone loss is directly related with disease severity.

Ultrasonography is a convenient and inexpensive imaging modality for the evaluation of the parathyroid gland with a sensitivity and specificity of 98% and 88%, respectively (24). The classical gray-scale imaging features include oval or lobulated extra-thyroidal hypoechoic lesions with a well-defined margin. In the present study, classical PAs had mostly hypoechoic appearances whereas APA and carcinomas had more isoechoic or cystic appearances. In addition, cystic change was an independent predictor for aggressive disease. There are scarce data in the literature concerning the prevalence and relevance of atypical ultrasound features for the evaluation of parathyroid lesions. Our study supports a previous report, which suggested that parathyroid lesions with atypical imaging features are associated with a higher incidence of malignancy (25). Moreover, in our study, the AUC for the longest adenoma diameter measured by ultrasonography was statistically significant, and the best cut-off value for predicting aggressive disease was 21.5 mm. In a previous report, the cut-off value for PC was 3 cm (26), while in another study the median tumor size at the time of diagnosis in 286 cases of PC treated in the USA from 1985 to 1995 was approximately 3.3 cm (27).

In our study the incidence of PC was higher than reported in the literature. Although parathyroid carcinoma (PC) is an uncommon finding, accounting for only 1-2% of patients with primary hyperparathyroidism (PHPT); a relatively higher incidence has been reported in Italy and Japan, in consistent with our study (28,29). Another possible etiology that can explain the higher incidence may be the fact that our hospital is a tertiary center receiving more complicated cases who are candidates for surgery. Moreover, the possible effects of radiation exposure after the Chernobyl disaster might have contributed to the higher incidence of cancers. Case reports and retrospective identification of several cases of parathyroid carcinoma in patients exposed to radiation have appeared in the literature in the last three decades supporting our hypothesis (30).

In this study we found that certain laboratory and clinical parameters were predictive for presence of aggressive disease. However we also observed that even mild clinical and laboratory disease may be associated with parathyroid carcinoma and benign disease could be found in large lesions with severe clinical picture. Similarly, Agarwal and cols. showed severe clinical features such as palpable parathyroid tumor, advanced skeletal and renal manifestations, and very high serum calcium and parathyroid hormone levels are common in Indian PHPT patients although only few have PC (31). Moreover Sulaiman and cols. showed that most of larger parathyroid lesions were benign PAs associated with specific genomic features (32).

A limitation of our study was that histopathology was not interpreted by one pathologist only, and ultrasonography was performed by two different endocrine specialists. Moreover, the number of patients diagnosed with carcinoma was small.

In conclusion, the clinical distinction between classical adenoma and aggressive parathyroid disease is of critical importance to determine the appropriate extent of resection and follow-up. Preoperatively high PTH, ALP, and UCa levels and large lesions with isoechoic or cystic appearances may be predictive of atypical adenoma or carcinoma in patients being evaluated for PHPT. In such cases, surgeons may prefer en bloc parathyroidectomy to minimally invasive surgery. It should also be kept in mind that those results are specific for our population, need to be verified in different populations and meanwhile the results should be interpreted with caution.
